# Integrated Transcriptome and Metabolome Analysis Provides Insights into the Low-Temperature Response in Sweet Potato (*Ipomoea batatas* L.)

**DOI:** 10.3390/genes16080899

**Published:** 2025-07-28

**Authors:** Zhenlei Liu, Jiaquan Pan, Sitong Liu, Zitong Yang, Huan Zhang, Tao Yu, Shaozhen He

**Affiliations:** 1 Agricultural College, China Agricultural University, Beijing 100107, China; liuzhenlei@cau.edu.cn (Z.L.); zhanghuan1111@cau.edu.cn (H.Z.); 2 Tuber Division, Crop Research Institute, Liaoning Academy of Agricultural Sciences, Shenyang 110095, China; pjqamy1001@163.com (J.P.); 15909824109@163.com (S.L.); yangzitong9870@163.com (Z.Y.)

**Keywords:** sweet potato, low temperature stress, transcriptome, metabolome

## Abstract

Background/Objectives: Sweet potato is a tropical and subtropical crop and its growth and yield are susceptible to low-temperature stress. However, the molecular mechanisms underlying the low temperature stress of sweetpotato are unknown. Methods: In this work, combined transcriptome and metabolism analysis was employed to investigate the low-temperature responses of two sweet potato cultivars, namely, the low-temperature-resistant cultivar “X33” and the low-temperature-sensitive cultivar “W7”. Results: The differentially expressed metabolites (DEMs) of X33 at different time stages clustered in five profiles, while they clustered in four profiles of W7 with significant differences. Differentially expressed genes (DEGs) in X33 and W7 at different time points clustered in five profiles. More DEGs exhibited continuous or persistent positive responses to low-temperature stress in X33 than in W7. There were 1918 continuously upregulated genes and 6410 persistent upregulated genes in X33, whereas 1781 and 5804 were found in W7, respectively. Core genes involved in Ca^2+^ signaling, MAPK cascades, the reactive oxygen species (ROS) signaling pathway, and transcription factor families (including bHLH, NAC, and WRKY) may play significant roles in response to low temperature in sweet potato. Thirty-one common differentially expressed metabolites (DEMs) were identified in the two cultivars in response to low temperature. The KEGG analysis of these common DEMs mainly belonged to isoquinoline alkaloid biosynthesis, phosphonate and phosphinate metabolism, flavonoid biosynthesis, cysteine and methionine metabolism, glycine, serine, and threonine metabolism, ABC transporters, and glycerophospholipid metabolism. Five DEMs with identified Kyoto Encyclopedia of Genes and Genomes (KEGG) pathways were selected for correlation analysis. KEGG enrichment analysis showed that the carbohydrate metabolism, phenylpropanoid metabolism, and glutathione metabolism pathways were significantly enriched and played vital roles in low-temperature resistance in sweet potato. Conclusions: These findings contribute to a deeper understanding of the molecular mechanisms underlying plant cold tolerance and offer targets for molecular breeding efforts to enhance low-temperature resistance.

## 1. Introduction

As one of the most important forms of abiotic stress, low temperatures pose a multifaceted threat to crop growth and yield, resulting in agricultural economic losses and production risks [1]. Chilling injury (0 to 15 °C) and freezing injury (below 0 °C) are the two main forms of low-temperature stress. Chilling injury primarily affects plants by reducing membrane fluidity and disrupting metabolic activities, while freezing injury causes structural damage to cells through the formation of ice crystals [2,3]. In general, low temperature affects a series of physiological and metabolic processes in plants, including cellular integrity, metabolic processes, and growth dynamics.

To cope with low-temperature stress, plants have evolved a series of adaptive mechanisms, including adaptive changes in the membrane system, the accumulation of osmoprotectants, the activation of the antioxidant defense system, and hormonal regulation [4]. Research has identified hundreds of metabolites that participate in the low-temperature regulatory network. Amino acids, sugars, carbohydrates, lipids, and enzymes are widely identified and play roles in stabilizing proteins and membrane structures and mitigating oxidative damage induced by low temperatures [5,6]. In addition to physiological and biochemical adjustments, plants also display a molecular mechanism of resistance to low-temperature stress through inducing the expression of several cold-related genes. Low-temperature-responsive genes produce protective proteins, such as COR-polypeptides (cold-regulated proteins) and antifreeze proteins (AFPs) [7]. The G-protein regulator chilling tolerance divergence 1 (COLD1), in combination with rice G-protein a subunit 1 (RGA1), regulates the cold-induced influx of Ca^2+^ to confer cold sensing in rice [8]. Several transcription factors (TFs) are reported to be involved in low-temperature stress responses, such as C-repeat binding factors (CBFs) [9], basic helix loop-helix (bHLH) [10], NAC [11], and MYB [12]. Additionally, genes related to membrane lipid metabolism, osmotic regulation, and antioxidant systems have also been shown to contribute significantly to withstanding low-temperature stress [13].

Sweet potato (*Ipomoea batatas* L.) is an important food and economic crop widely cultivated around the world, making a significant contribution to global food security [14]. The global cultivation area of sweet potatoes is approximately 8 million hectares, primarily in tropical and subtropical regions of Asia, Africa, and the Americas. China is the world’s largest producer of sweet potatoes, with an annual output exceeding 50 million tons, accounting for more than 50% of the global total production [15]. However, as a tropical and subtropical crop, sweet potato is relatively sensitive to low temperatures, and its growth and yield are susceptible to low-temperature stress. When the temperature drops below 15 °C, the growth of sweet potatoes slows significantly or even halts. As the temperature approaches 0 °C, sweet potato plants suffer from frost damage, leading to the destruction of cellular structures and ultimately causing plant death [16]. Several important genes associated with low-temperature tolerance have been identified in sweet potato. These genes help increase low-temperature stress tolerance by activating cold-responsive pathways, enhancing antioxidant defenses, and stabilizing cell membranes. Notable examples include *IbCBF3*, *IbHLH79*, *IbMPK3,* and *IbLfp* [17,18,19,20].

Metabolomic analysis has unveiled the intricate metabolic networks that plants activate in response to low-temperature stress. Under low-temperature stress, plants engage a suite of metabolic pathways, including glycolysis, the tricarboxylic acid (TCA) cycle, amino acid metabolism, and antioxidative metabolism, to preserve cellular homeostasis and energy equilibrium. The synergistic interplay of these pathways forms a critical mechanism through which plants combat the challenges posed by low temperatures. Metabolomics provides essential insights into the metabolic underpinnings of variations in cold hardiness among plants. Research has demonstrated that metabolites such as galactinol, raffinose, and pinitol are closely linked to low-temperature tolerance in plants [21,22,23]. Comparing the metabolic profiles of cultivars with differing levels of cold resistance enables the identification of key metabolic pathways and regulatory nodes associated with cold tolerance, such as soluble sugars, amino acids, and plant hormones. Additionally, two transcriptome assembly methods, de novo assembly and reference assembly, provided a more comprehensive dataset and revealed significant changes in the expression of numerous genes, including TFs, signal transduction-related genes, and metabolism-related genes under environmental stresses, which enabled an unbiased interpretation of the outcomes [24,25]. These genes collectively form a complex regulatory network involved in the response of plants to low-temperature stress [26,27]. In summary, these findings contribute to a deeper understanding of the molecular mechanisms underlying plant cold tolerance and offer targets for molecular breeding efforts to enhance low-temperature resistance.

The northern spring potato region, as one of China’s important sweet potato production areas, is more susceptible to low-temperature stress due to geographical and seasonal factors. This severely hinders sweet potato seedling cultivation and planting, limiting its production and application [28]. In our previous study, we collected 84 sweet potato cultivars around the world and identified their low-temperature stress tolerance ability, including survival rate, electrical conductivity, antioxidant enzyme activity and osmoregulation substance. We have found cv. X33 is a low-temperature stress-tolerant cultivar, while W7 is low-temperature sensitive [29]. The present study aimed to explore the key genes and metabolic pathways activated in response to low-temperature stress based on a combination of transcriptome and metabolism analysis using X33 and W7. Differentially expressed genes (DEGs), differentially expressed metabolites (DEMs), and the vital regulatory pathway were identified, providing important insights into the mechanisms underlying low-temperature tolerance in sweet potato.

## 2. Materials and Methods

### 2.1. Plant Materials and Cold Stress Treatment

Two sweet potato varieties, the cold-tolerant cultivar cv. X33 and the cold-sensitive cultivar cv. W7 was selected for the experiments. The sweetpotato cv. X33 was bred by Xuzhou Academy of Agricultural Sciences and cv. W7 was bred by Wanzhou Academy of Agricultural Sciences. The seedlings of the two sweet potato cultivars were planted in the artificial climate room of the potato research laboratory, Liaoning Academy of Agricultural Sciences. Healthy and fully developed 2-week-old sweet potato plantlets were placed in hydroponics devices for 3 days under a 16/8 h light/dark cycle at 25 °C until root germination and exposed to low-temperature treatment at 4 °C. Samples were collected at 0 h (control), 3 h, and 24 h after treatment, with three biological replicates. The samples were photographed, and the 3rd to 4th leaves from the bottom of the plants were flash-frozen in liquid nitrogen and stored at −80 °C for transcriptomic and metabolomic sequencing. The survival rate was counted in 2021 and 2022 using 100 seedlings of each sweet potato variety under the 4 °C treatment.

### 2.2. Transcriptome Analysis

Total RNA from the 3rd to 4th leaves from the top of sweet potato was extracted using RNA extraction kit (Generay, Beijing, China). NanoDrop quantifies concentration and purity (A260/A280 ratio ≈ 2.0), while gel electrophoresis validates size and integrity. First-strand cDNAs were produced by reverse transcription using a random hexamer primer, and then second-strand cDNAs were synthesized with end repair and dA-Tailing. First-strand cDNAs were produced by reverse transcription using a random hexamer primer, and then second-strand cDNAs were synthesized with end repair and dA-Tailing. Sequencing adapter ligation was performed, followed by DNA magnetic bead purification, and fragment selection after ligation was completed to yield a library with 250–350 bp insert fragments.

The cDNA libraries prepared from samples of sweet potato plants exposed to low-temperature stress for 0 h, 3 h, and 24 h, designated as X0, X3, X24 for cv. X33, respectively, and W0, W3, and W24 for cv. W7, respectively, were constructed and sequenced on the Illumina Hiseq 2000 platform according to the manufacturer’s instructions (Illumina, San Diego, CA, USA). After removing low-quality reads and adaptors with ShortRead Package, qualified clean reads were assembled using Trinity software (version 2.15.1) and mapped to the sweet potato reference genome sequence Taizhong 6 accessed on 30 August 2024 (http://sweetpotao.com/) in Tophat2.0 software.

The samples were subjected to principal component analysis (PCA), cluster heatmap analysis, and correlation analysis using the R package. The fragments per kilobase of transcript per million mapped reads (FPKM) values were employed to estimate gene expression levels. DEGs among groups were identified using the DESeq R package (1.10.1) based on the negative binomial distribution model with |log_2_fold change (FC)| ≥ 1 (|Fold Change| ≥ 2) and false discovery rate (FDR) ≤ 0.01 [30]. EnhancedVolcano Package with significance thresholds set at *p* < 0.05 and |log_2_FC| > 1 was used to draw volcano plot. The analysis of DEG enrichment in Gene Ontology (GO) terms was performed using the origGO web-based program (http://systemsbiology.cau.edu.cn/, accessed on 30 August 2024) at *p* ≤0.05 [31,32], and Kyoto Encyclopedia of Genes and Genomes (KEGG) pathway analysis [33] was conducted in the KOBAS2.0 web-based program (http://www.genome.jp/kegg, accessed on 30 August 2024) at *p* value ≤ 0.01 [34].

### 2.3. Metabolomics Analysis

Using vacuum freeze-drying technology, place the biological samples in a lyophilizer (Scientz-100F, Ningbo, China), then grind (30 Hz, 1.5 min) the samples to powder form by using a grinder (MM 400, Retsch, Duesseldorf, Germany). Next, weigh 50 mg of sample powder using an electronic balance (MS105DΜ, Shanghai, China) and add 1200 μL of −20 °C pre-cooled 70% methanolic aqueous internal standard extract (less than 50 mg added at the rate of 1200 μL extractant per 50 mg sample). Vortex once every 30 min for 30 s, for a total of 6 times. After centrifugation (rotation speed 12,000 rpm, 3 min), the supernatant was aspirated, and the sample was filtered through a microporous membrane (0.22 μm pore size) and stored in the injection vial for liquid chromatography–tandem mass spectrometry (LC-MS/MS) analysis. Briefly, 0.1 g sample of powder was added to 80% methanol and centrifuged at 15,000× *g* and 4 °C for the absorption of the extractives. The extractives were further diluted to 53% methanol with liquid LC-MS/MS grade water and injected into the LC-MS/MS system for positive and negative ion mode analyses. The primary and secondary mass spectrometry data in MSE mode were collected using MassLynx software (version 4.2, Waters Crop., Milford, CT, USA) and analyzed in Progenesis QI software (version 3.0) (Waters Crop., Milford, CT, USA). The metabolites were identified using the METLIN online database (Biomaker Technologies Co., Ltd. (Beijing, China)) with the mass number deviation within 100 ppm [35,36].

To ensure instrument stability, we perform simultaneous testing of the test samples mixed at equal ratios with quality control (QC) samples to ensure the stability of liquid chromatography-mass spectrometry system. The repeatability of QC samples is evaluated using PCA plots and QC sample correlation plots. A QC sample correlation coefficient >0.8 indicates stable instrument performance during the experiment, meeting quality control requirements. Additionally, unit variance scaling (UV) normalization is applied for PCA analysis and cluster analysis, and in OPLS-DA (Orthogonal PLS-DA) analysis, zero-centered (Ctr) processing is implemented.

The DEMs among different comparison groups were detected based on the variable importance in projection (VIP) values obtained using partial least squares-discriminant analysis (PLS-DA) and the univariate statistical analysis of the *t* test *p* value with a threshold of Log2FC ≥ 1, VIP > 1, and *p* < 0.05. The DEM analysis methods were similar to those employed to analyze DEGs.

### 2.4. Integrated Transcriptome and Metabolome Analysis

Networks were visualized using chiplots Network Plot module (https://www.chiplot.online/) with a force-directed layout [37]. Significance thresholds were set at the Pearson correlation coefficient (PCC) > |0.8| at *p* < 0.05.

## 3. Results

### 3.1. Physiological Responses of X33 and W7 Sweet Potatoes to Low-Temperature Stress

**Figure 1:**
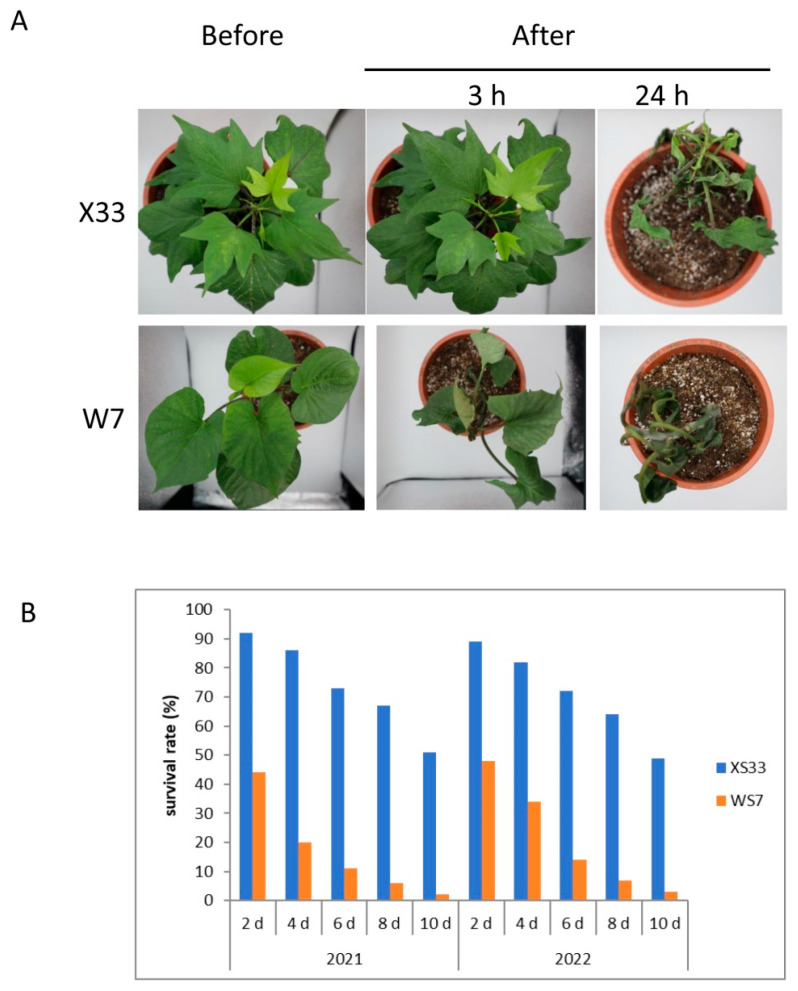
Physiological performance of cv. X33 and W7 sweet potato genotypes. (**A**) Morphological changes of X33 and W7 under low-temperature stress. (**B**) Survival rate of X33 and W7 under low-temperature stress. Notably, 100 seeds of each variety were used for survival rate tests in 2021 and 2022.

To analyze the variation of sweet potato tolerance to low temperature, cv. X33 (low-temperature-tolerant genotype) and cv. W7 (sensitive to low temperature) with similar height (19.04 cm–22.52 cm) with 91.06–91.51% rate of water content were selected according to previous studies. X33 displayed better cold tolerance than W7, with only slight wilting under 3 h after low-temperature stress. Under low-temperature stress for 24 h, leaves of both varieties exhibited wilting and dehydration, but the symptoms were much more severe in W7 (Figure 1A). There was a significant difference in the rate of water content between the two cultivars under low-temperature stress treatment. The rate of water content in X33 and W7 was 86.32% and 46.78% on average after 3 h treatment, and 53.21% and 24.51% on average after 24 h treatment, respectively. The survival rates for two consecutive years were examined to further illustrate the distinct low-temperature response between the two genotypes. As shown in Figure 1B, about half of W7 seedlings did not survive after 2 days of treatment, while more than 90% of X33 seedlings survived.

### 3.2. Metabolic Profiling and DEMs Involved in Low-Temperature Responses Between Two Sweet Potato Varieties

**Figure 2:**
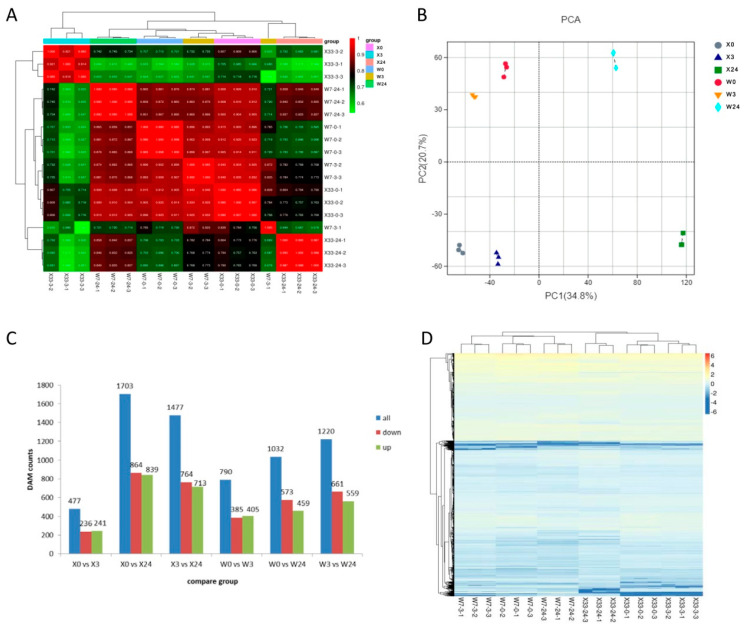
Metabolic profiling of X33 and W7 sweet potato genotypes in response to low-temperature stress. (**A**) Correlation heatmap among different groups. (**B**) Principal component analysis (PCA) based on metabolomic data. The X−axis represents the first principal component (PC1) and the Y−axis represents the second principal component (PC2). Different groups with three biological repeated samples were marked with squares, diamonds, and triangles on the PCA plot. (**C**) Total number of upregulated and downregulated differentially expressed metabolites (DEMs). (**D**) Hierarchical cluster analysis for metabolomics profiles based on the fold change *p* < 0.05 and log_2_Foldchange > 1.5.

To construct a systematic profile of metabolic changes induced by low-temperature stress in sweet potato, an untargeted metabolome analysis was conducted under normal conditions and low-temperature stress. In total, 3940 metabolites were detected and classified as amino acids, lipids, membrane transport, cofactors, vitamins, terpenoids, polyketides, and secondary metabolites. Hierarchical cluster analysis and PCA were performed to better understand the differences in metabolic profiles in response to low-temperature stress between X33 and W7. As shown in Figure 2A, hierarchical clustering indicated that metabolic data for X33 obviously differed from W7, and the metabolic data from the short-term low-temperature treatment (3 h) were clearly separated from the results of the relatively long-term low-temperature treatment. Volcano plots were also drawn to further visualize the differential gene expression between low-temperature stress and normal conditions in the X33 and W7 (Supplementary Figure S2).

PCA showed that the assignment of X0 and X3 was similar, as well as W0 and W3, which suggested that fewer metabolic changes occurred between 0 and 3 h of low-temperature stress in sweet potato. Low-temperature stress for 24 h resulted in clear separation compared to 0 h and 3 h of low-temperature stress treatment, indicating good intra-group reproducibility and high similarity among the sample data, while demonstrating clear differentiation between groups (Figure 2B). The DEMs were identified based on pairwise comparisons of metabolism datasets obtained from X33 and W7 plants at the different time points under low-temperature stress. The number of DEMs under normal conditions was compared to the number of DEMs under low-temperature stress at each time point. As shown in Figure 2C, 477 DEMs were obtained from the comparison of X0 vs. X3 (236 upregulated DEMs and 241 downregulated DEMs), 1703 DEMs from X0 vs. X 24 (864 upregulated DEMs and 839 downregulated DEMs), 1477 DEMs from X3 vs. X24 (764 upregulated DEMs and 713 downregulated DEMs), 790 DEMs from W0 vs. W3 (385 upregulated DEMs and 405 downregulated DEMs), 1032 DEMs from X0 vs. W24 (573 upregulated DEMs and 459 downregulated DEMs), and 1220 DEMs from W3 vs. W24 (661 upregulated DEMs and 559 downregulated DEMs). The upregulated DEMs obtained from the comparison of X0 vs. X3, X0 vs. X24, W0 vs. W3, and W0 vs. W24 were enriched in KEGG pathways of “phenylpropanoid biosynthesis”, “biosynthesis of cofactors”,” biosynthesis of alkaloids derived from shikimate pathway”, and “biosynthesis of type II polyketide products”, respectively. The downregulated DEMs were enriched in “biosynthesis of alkaloids derived from shikimate pathway” obtained from the groups of X0 vs. X3 and X0 vs. X24, and “biosynthesis of cofactors” obtained from the groups of W0 vs. W3 and W0 vs. W24, respectively.

**Figure 3:**
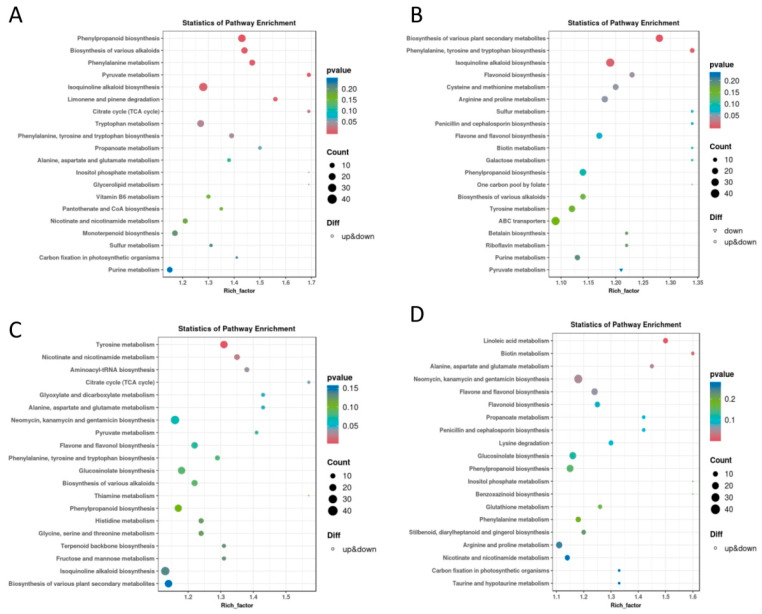
Kyoto Encyclopedia of Genes and Genomes (KEGG) pathway enrichment analysis of differentially expressed metabolites (DEMs) related to low-temperature stress. The sweet potato genotype X33 under low-temperature treatment for 3 h (**A**) and 24 h (**B**) compared to normal conditions, and the genotype W7 under low-temperature treatment for 3 h (**C**) and 24 h (**D**) compared to normal conditions. The most enriched relevant terms are shown in the plots.

Additionally, to better reveal the functions of these DEMs, all DEMs were mapped to KEGG pathways. DEMs in X33 plants were assigned to the KEGG pathways of “phenylpropanoid biosynthesis”, “biosynthesis of various alkaloids” and “phenylalanine metabolism” for the short-term stress treatment (Figure 3A), and to the KEGG pathways of “biosynthesis of various plant secondary metabolites”, “phenylalanine, tyrosine and tryptophan biosynthesis”, and “isoquinoline alkaloid biosynthesis” for the long-term stress treatment (Figure 3B). For W7 plants, DEMs were enriched in KEGG pathways including “tyrosine metabolism”, “nicotinate and nicotinamide metabolism”, and “aminoacyl-tRNA biosynthesis” for the short-term stress treatment (Figure 3C), and in the KEGG pathways of “linoleic acid metabolism”, “biotin metabolism”, and “alanine, aspartate and glutamate metabolism” for the long-term stress treatment (Figure 3D). The DEMs involved in the KEGG pathways at the same time points between the two varieties were notably different, which indicates that X33 and W7 may display distinct molecular mechanisms in response to low-temperature stress.

**Figure 4:**
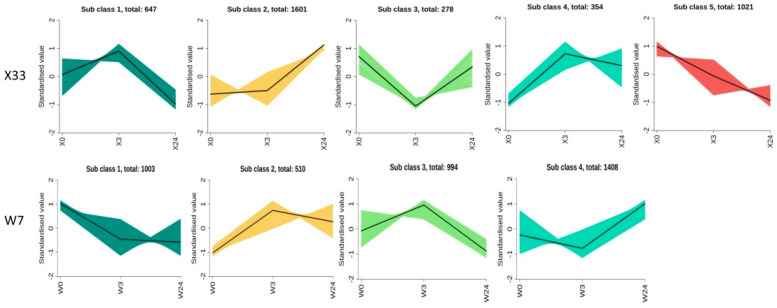
Change trends of the DEMs across three time points in the two sweet potato genotypes. Among the change trends, DEMs continuously increased, remained unchanged first and then increased, increased and then decreased, decreased and then increased, continuously decreased, and remained unchanged first and then decreased. In each frame, color lines represent the gene expression pattern, while black lines represent the expression tendency of all genes. The number of metabolisms belonging to each pattern is given above the frame.

Moreover, the DEMs of X33 at different time stages clustered in five profiles, while they clustered in four profiles of W7 with significant differences (*p* < 0.01) (Figure 4). Two typical cluster moderns were only in X33, including 1601 continuously upregulated metabolisms and 1021 persistently downregulated metabolisms. There were 2604 metabolisms upregulated at 3 h of X33, and then 647 metabolisms downregulated with lower relative contents at 24 h compared with 0 h. In comparison, there were 1504 metabolisms upregulated at 3 h of W7, and then 994 metabolisms downregulated with lower relative contents at 24 h compared with 0 h. Taken together, the different DEMs between the two sweet potato cultivars showed the distinct response to low-temperature stress.

### 3.3. Core Metabolites in Response to Low-Temperature Stress

**Figure 5:**
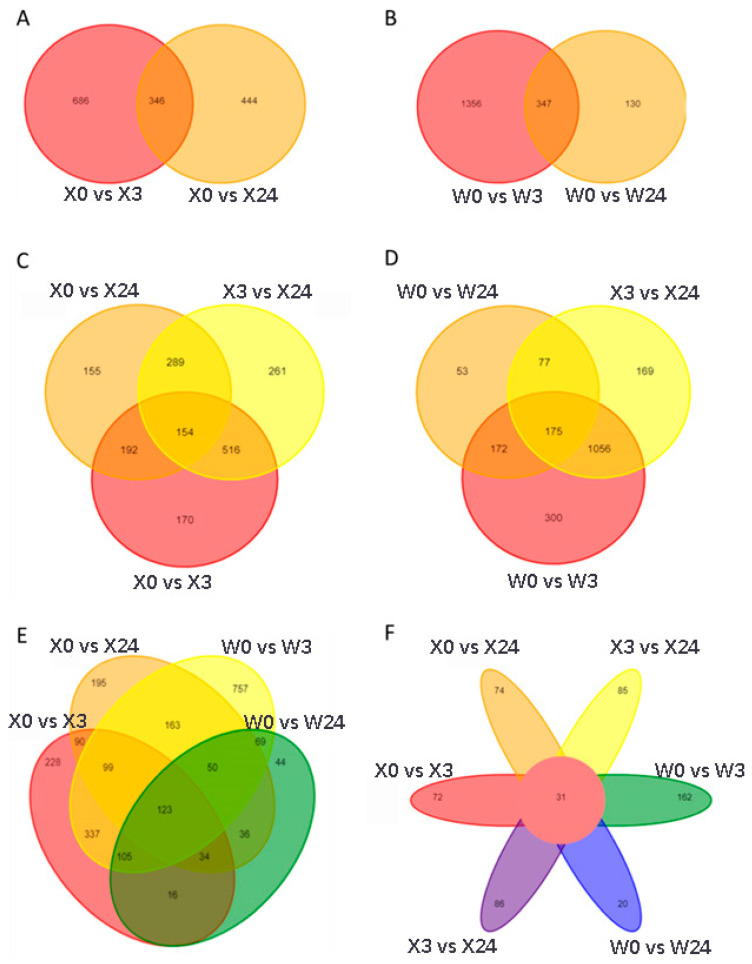
Venn diagram of differentially expressed metabolites (DEMs) in different comparison groups. Venn diagrams showing the shared DEMs at different time points in X33 (**A**,**C**), W7 (**B**,**D**), and both X33 and W7 (**E**,**F**).

Common DEMs were identified between both sweet potato varieties and at different time points within each cultivar under low-temperature stress conditions (Figure 5). There were 346 DEMs in the overlapping section of X0 vs. X3 and X0 vs. X24 (Figure 5A) and 154 DEMs between the overlapping section of X0 vs. X3, X0 vs. X24, and X3 vs. X24 (Figure 5C) in X33, while 347 DEMs were in the overlapping section of W0 vs. W7 and W0 vs. W24 (Figure 5B) and 175 DEMs in the overlapping section of W0 vs. W3, W0 vs. W24, and W3 vs. W24 (Figure 5D) in W7, respectively. In addition, Venn diagram analysis revealed the unique and common DEMs among different treatment groups between X33 and W7. There were 123 common DEMs in the overlapping section of X0 vs. X3, X0 vs. X4, W0 vs. W3, and W0 vs. W24 shown in Figure 5E, and 31 common DEMs in the overlapping section of X0 vs. X3, X0 vs. X4, W0 vs. W3, and W0 vs. W24 shown in Figure 5F. As shown in Table 1, the 31 common core DEMs were selected for further analysis. The KEGG analysis of these DEMs revealed that these metabolites mainly belonged to isoquinoline alkaloid biosynthesis, phosphonate and phosphinate metabolism, flavonoid biosynthesis, cysteine and methionine metabolism, glycine, serine, and threonine metabolism, ABC transporters, and glycerophospholipid metabolism.

### 3.4. General Description of Transcriptome Data

**Figure 6:**
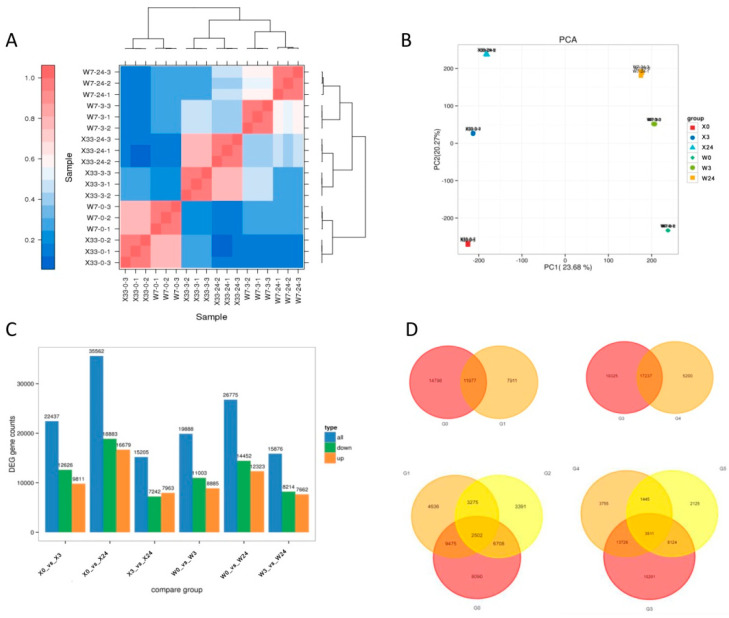
Transcription profiling of sweet potato genotypes X33 and W7 in response to low-temperature stress. (**A**) Correlation heatmap among different groups. (**B**) Principal component analysis (PCA) based on metabolomic data. The X-axis represents the first principal component (PC1) and the Y-axis indicates the second principal component (PC2). (**C**) Total number of upregulated and downregulated differentially expressed genes (DEGs). (**D**) Venn diagram of DEGs in different comparison groups.

A total of 729.07 Mb of raw reads for 18 samples were generated through RNA sequencing, and libraries were constructed using the clean reads. All of the raw reads were deposited in the NCBI SRA database (accession number: PRJNA1266597). The Q20 and Q30 values of each cDNA library were greater than 97% and 92%, respectively. In total, 73% or more of clean reads were successfully aligned to the sweet potato reference genome (Table S1). To dissect the changes in gene expression induced by low-temperature stress, global transcriptome profiles were constructed. The correlation assessment and PCA showed that the replicates for each treatment clustered together. PCA explained 55.5% of the total variance, including 23.68% for PC1 and 20.27% for PC2 (Figure 6B). The isolation of genotype was distinguished by PC1, while different low-temperature stress time points were separated by PC2.

**Figure 7:**
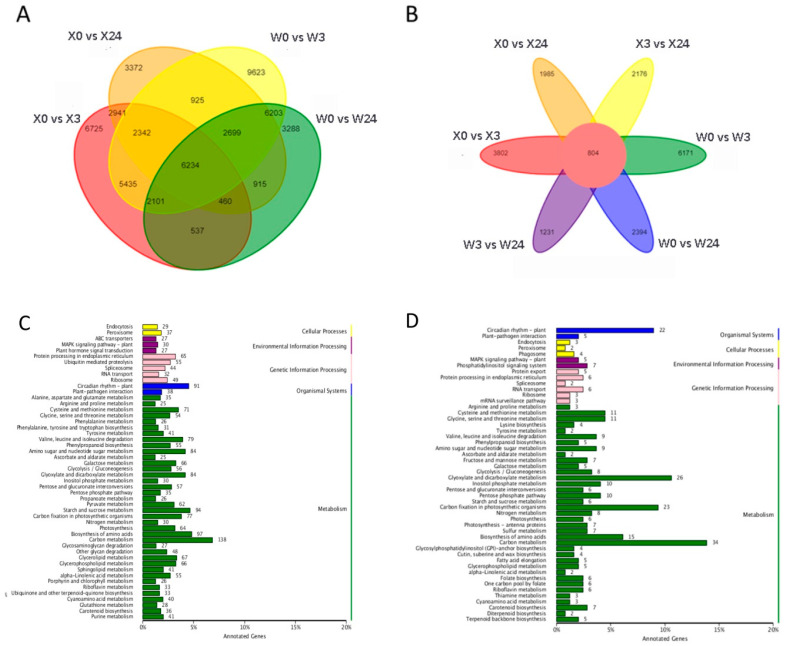
Common differentially expressed gene (DEG) analysis between X33 and W7 in response to low-temperature stress. (**A**) Venn diagrams showing the shared DEGs at different time points among the groups X0 vs. X3, X0 vs. X24, W0 vs. W3, and W0 vs. W24. (**B**) Venn diagrams showing the shared DEGs among the groups X0 vs. X3, X0 vs. X24, W0 vs. W3, W0 vs. W24, X3 vs. X24, and W3 vs. W24. (**C**,**D**) Related Kyoto Encyclopedia of Genes and Genomes (KEGG) pathways were analyzed for the shared DEGs in Figure 6A,B. The most enriched relevant terms are shown in the plots.

As shown in Figure 6C,D, DEGs were identified based on pairwise comparisons of transcriptome datasets from the two sweet potato varieties at three time points. In total, there were 22,437 DEGs (12,626 upregulated and 9811 downregulated DEGs) at 3 h (vs. 0 h) and 35,562 DEGs (18,883 upregulated and 16,679 downregulated DEGs) at 24 h (vs. 0 h) in X33, whereas only 19,888 DEGs (11003 upregulated and 8885 downregulated DEGs) at 3 h (vs. 0 h) and 26,775 DEGs (14,452 upregulated and 12,323 downregulated DEGs) were identified in W7 at 24 h (vs. 0 h). In addition, 6234 common DEGs were identified in the overlapping section among X0 vs. X3, X0 vs. X24, W0 vs. W3, and W0 vs. W24 (Figure 7A), while 804 common DEGs were identified in the overlapping section among X0 vs. X3, X0 vs. X24, X3 vs. X24, W0 vs. W3, W0 vs. W24, and W3 vs. W24 (Figure 7B). KEGG classification analysis was implemented to determine the potential function of DEGs in response to low-temperature stress. The 6234 common DEGs in the two sweet potato cultivars were enriched in the first three pathways “Carbon metabolism”, “starch and sucrose metabolism”, and “circadian rhythm-plant” (Figure 7C). The 804 common DEGs were involved in similar KEGG pathways with Figure 6C (Figure 7D). The pathway of “carbon metabolism” displayed the most enrichment, followed by the “clyoxylate and dicarboylate” and “circadian rhythm-plant” pathways.

**Figure 8:**
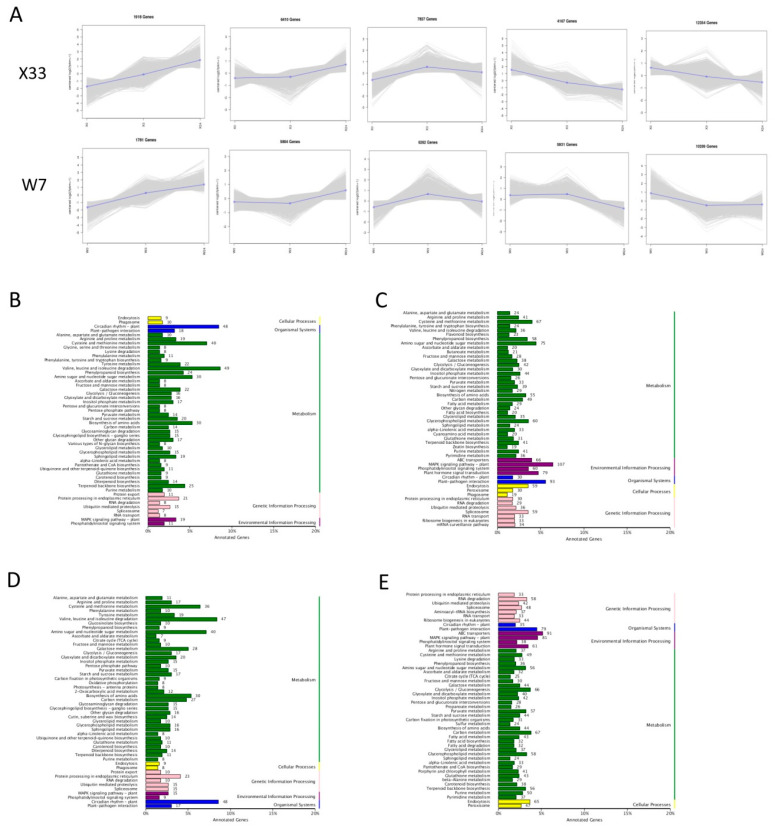
Differentially expressed gene (DEG) expression patterns and related Kyoto Encyclopedia of Genes and Genomes (KEGG) pathway analysis in both X33 and W7 in response to low-temperature stress. Change trends of the DEGs across three time points in the two sweet potato genotypes (**A**). Among the change trends, DEGs continuously increased, remained unchanged first and then increased, increased and then decreased, continuously decreased, and remained unchanged first and then decreased. In each frame, gray lines represent the gene expression pattern, while blue lines represent the expression tendency of all genes. The number of genes belonging to each pattern is given above the frame. The related KEGG pathways were analyzed, including the continuously increasing trends in X33 (**B**) and W7 (**D**) and the tendency to remain unchanged first and then increase in X33 (**C**) and W7 (**E**).

The DEGs of X33 and W7 at different time stages clustered in five profiles, with significant differences (*p* < 0.01) (Figure 8). It was clear that there were two identical increasing expression profiles in both sweet potato varieties. More DEGs exhibited continuous or persistent positive responses to low-temperature stress in X33 than in W7. There were 1918 continuously upregulated genes and 6410 persistently upregulated genes in X33, whereas 1781 continuously upregulated genes and 5804 persistently upregulated genes were detected in W7. KEGG pathway analysis was conducted to explore the potential functions of upregulated genes. In X33, the most abundant persistently upregulated genes belonged to “circadian rhythm”, and the most abundant persistently upregulated genes were enriched in “MAPK signaling system”, whereas in W7 were assigned to “valine, leucine, and isoleucine degradation” and “ABC transporter”. These results imply that differential gene expression patterns in X33 and W7 may be the basis for their different levels of tolerance to low-temperature stress.

### 3.5. Functional and Enrichment Analysis of Common DEGs

Common DEGs in X33 and W7 were identified to further investigate the mechanism of low-temperature tolerance in sweet potato (Table 2). Low temperatures reduce the fluidity of the cell membrane and cause modifications in the membrane and cytosolic Ca^2+^ flux, which is sensed by calcium channels or proteins located in the plasma membrane. As shown in Table 2, there were 10 DEGs encoding Ca^2+^-binding proteins, including 4 calcium-binding protein KRP1s, three calcium-binding protein CML19s, and 3 calmodulin-binding proteins. MAPK cascades (MAPKKK, MAPKK, and MAPK), serine/threonine-protein kinase (STK), leucine-rich repeat receptor-like kinase (LRR), and CBL-interacting protein kinase (CIPK) act as signal transduction molecules activated by the Ca^2+^/CaM signaling pathway. Several TFs together induce COR gene expression under low-temperature stress. In the present study, 59 DEGs encoding TFs were identified as AP2, MYB, WRPK, bHLH, NAC, and ZIP. Common stress-responsive genes were also identified, including pathogenesis- and peroxidase-related genes. In addition, crosstalk occurred between plant hormone signals and low-temperature signals. A total of 12 hormone-related DEGs were found to be associated with abscisic acid (ABA), jasmonic acid (JA), and ethylene (ETH) synthesis and the transduction pathways of various hormones.

### 3.6. Joint Analysis of DEMs and DEGs

**Figure 9:**
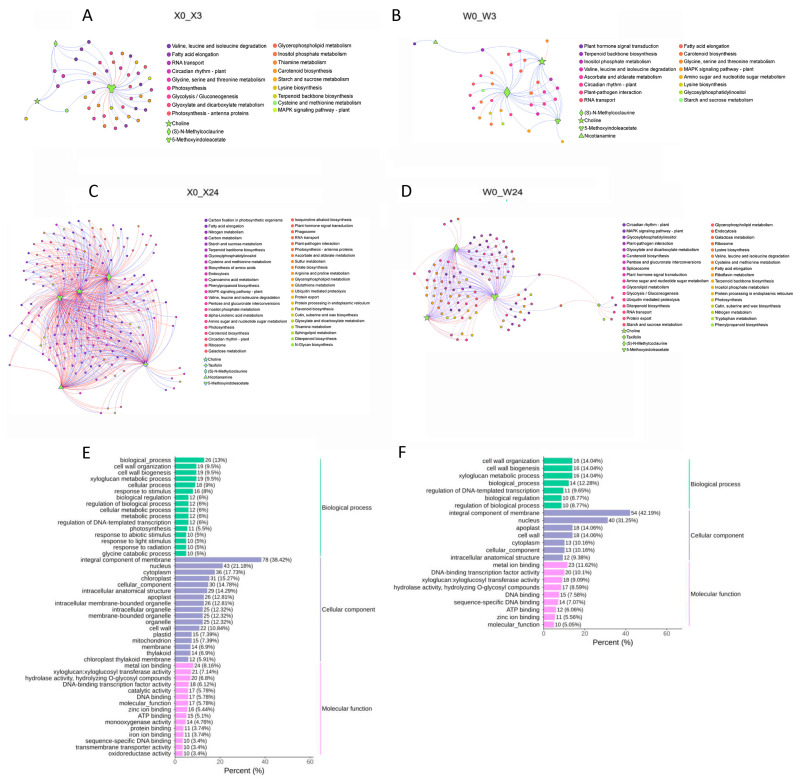
Correlation network analysis between differentially expressed metabolites (DEMs) and regulatory genes related to low-temperature stress. Analysis of X0 vs. X3 (**A**), W0 vs. W3 (**B**), X0 vs. X24 (**C**), and W0 vs. W24 (**D**). Five DEMs were selected from the common DEMs between X33 and W7 at different time points with definite KEGG pathways. The metabolites marked with numbers 1, 2, 3, 4, and 5 are 5-methoxyindoleacetate, choline, (S)-N-methylcoclaurine, nicotianamine, and taxifolin, respectively. Nodes represented metabolites and genes; edges indicated significant correlations, and positive/negative edges were retained. (**E**,**F**) Analysis of GO enrichment of the genes in X0 vs. X24 and in W0 vs W4, respectively. The most enriched relevant terms are shown in the plots.

Thirty-one common DEMs were identified at different time points between X33 and W7 under low-temperature conditions (Table 1). Five metabolic pathways with specific KEGG pathways were selected for correlation analysis. As shown in Figure 9, the correlation analysis was performed between five DEMs and their corresponding DEGs within four groups. The DEMs were 5-methoxyindoleacetate, choline, (S)-N-methylcoclaurine, nicotianamine, and taxifolin, which may contribute to low-temperature stress resistance. There were more correlated DEGs in the X33 groups compared to the W7 groups, which may indicate more complex low-temperature stress resistance mechanisms in X33 than in W7. Additionally, the DEGs within X0 vs. X24 and W0 vs. W24 selected by correlation analysis between DEM and DEGs were further subjected to GO category analysis to determine the specific GO terms related to the five key DAMs under low-temperature conditions (Figure 9E,F). Most GO terms in X0 vs. X3 (Figure 9E) are similar to W0 vs. W3 (Figure 9F). The highly enriched DEGs are associated with integral components of the membrane (GO:001602) and nucleus (GO:005634) in cellular components, cell wall organization (GO: GO:0071555) in biological processes, and metal ion binding (GO: GO:0046872) in molecular function. However, a few GO terms were enriched only in X0 vs. X3, not in W0 vs. W3. Including chloroplast (GO: GO:0009507) in cellular component and catalytic activity (GO: GO:0003824) in molecular function, which may result in the distinct ability to tolerate low-temperature stress between X3 and W7. Taken together, the GO enrichment analysis in this study provided important guidance to identify DEGs that functioned in key pathways that may be directly or indirectly involved in the low-temperature response of sweet potato.

### 3.7. Carbohydrate Metabolism in Response to Low Temperatures

**Figure 10:**
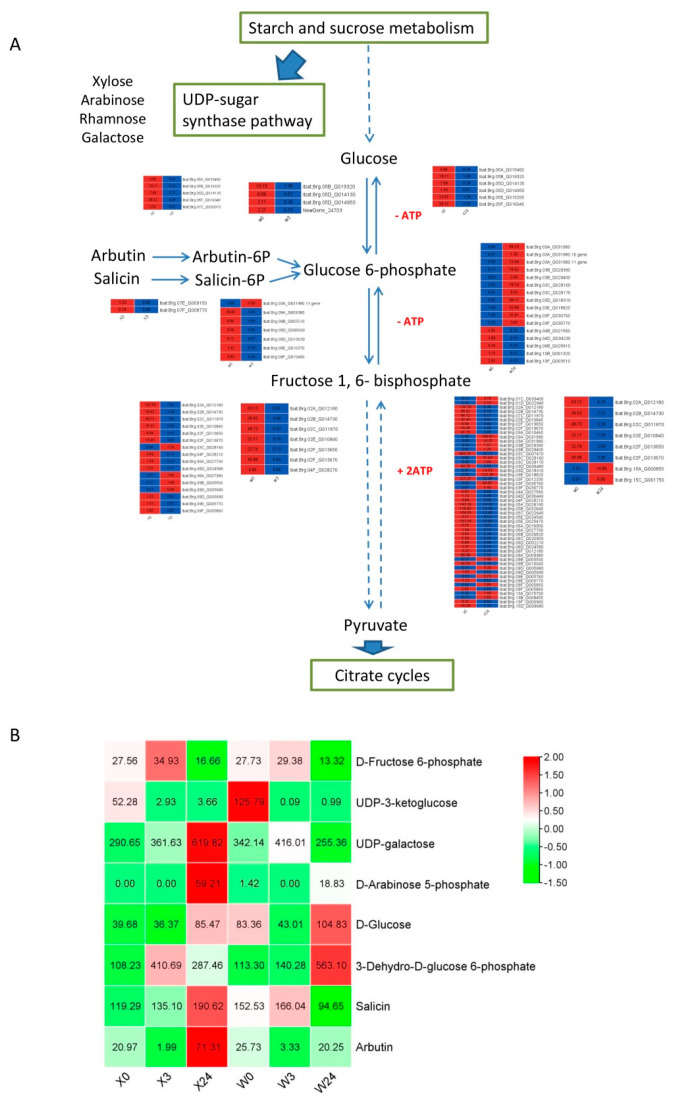
Adaptive changes involved in sugar metabolism in X33 and W7 under low-temperature stress. (**A**) Heatmap of DEGs involved in sugar metabolism. The solid arrow and dashed arrow represent one reaction and several reactions, respectively. The red and blue rectangles indicate the expression pattern (up-/downregulated) of DEGs based on the two comparisons, i.e., X0 vs. X3, X0 vs. X24, W0 vs. W3, and W0 vs. W24. (**B**) Heatmap of DEMs involved in sugar metabolism. The red and green rectangles indicate the related contents (up-/downregulated) of DEMs based on the two comparisons, i.e., X0 vs. X3, X0 vs. X24, W0 vs. W3, and W0 vs. W24.

Carbohydrate metabolism encompasses multiple pathways, including sucrose and starch synthesis, glycolysis/gluconeogenesis, the pentose phosphate pathway (PPP), and the TCA cycle [38]. According to the results of the present study, carbohydrate metabolism was significantly enriched in both metabolome and transcriptome data. Figure 10 depicts the changes in important metabolomes and genes. The contents of several soluble sugars, including galactose, arabinose, and glucose, were increased in both sweet potato cultivars under low-temperature stress (Figure 10B). However, the soluble sugar contents were much higher in X33 than in W7, such as UDP-galactose and D-arabinose 5-phosphate. Additionally, hexokinase and phosphofructokinase are two critical enzymes in the glycolytic pathway [39,40]. In the present study, there were six DEGs encoding hexokinase and seven DEGs encoding phosphofructokinase in X33, while four DEGs encoding hexokinase and 16 DEGs encoding phosphofructokinase were identified in W7. The relative content (response value) of fructose 6-phosphate in X33 was similar to that in W7 at the same time point, while the highest point of gluose-6-phosphate content in X33 is earlier than that in W7. Salicin and arbutin scavenge free radicals and alleviate oxidative stress damage [41]. In the present study, the salicin and arbutin response values were 190.62 and 71.31 in X33, respectively, while the salicin and arbutin response values of W7 were 94.65 and 20.25, respectively. The results showed that X33 performed better in accumulating some carbohydrates and involved genes and thus exhibited superior low-temperature resistance compared to W7.

### 3.8. Phenylalanine Metabolism in Response to Low Temperatures

**Figure 11:**
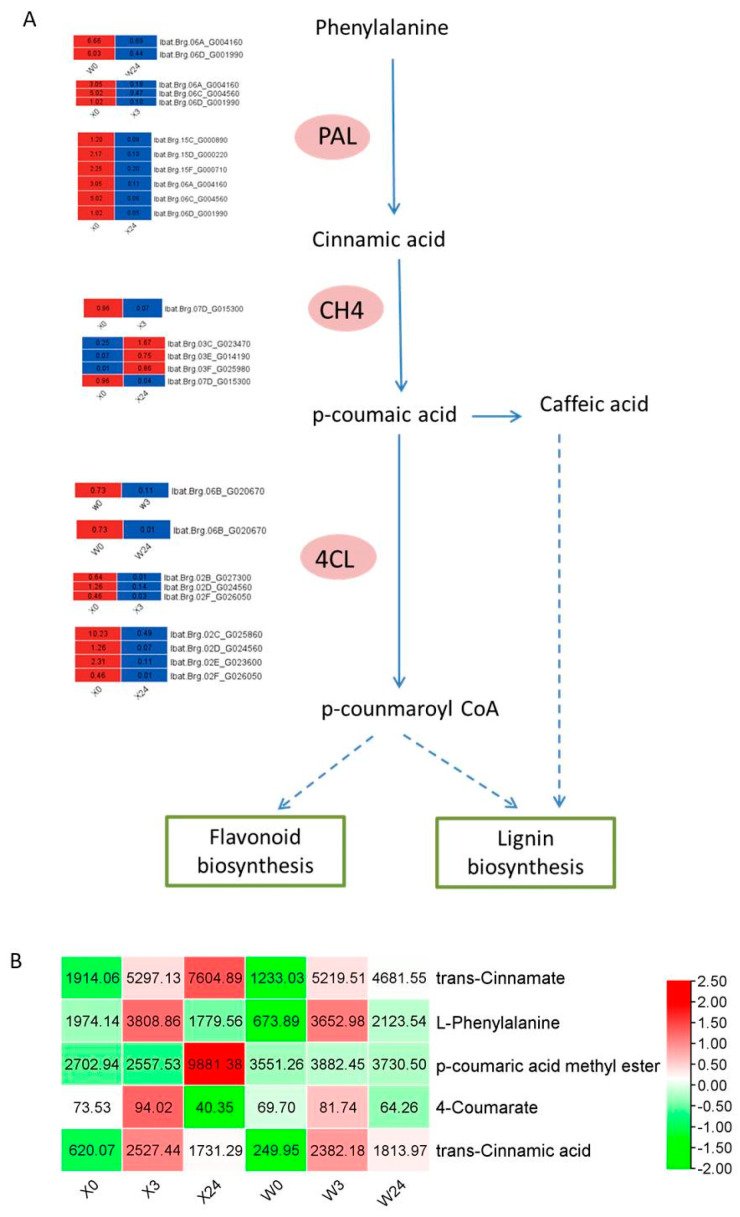
Adaptive changes involved in phenylalanine metabolism in X33 and W7 under low-temperature stress. (**A**) Heatmap of DEGs involved in phenylalanine metabolism. The solid arrow and dashed arrow represent one reaction and several reactions, respectively. The red and blue rectangles indicate the expression pattern (up-/downregulated) of DEGs based on the two comparisons, i.e., X0 vs. X3, X0 vs. X24, W0 vs. W3, and W0 vs. W24. (**B**) Heatmap of DEMs involved in phenylalanine metabolism. The red and green rectangles indicate the related contents (up-/downregulated) of DEMs based on the two comparisons, i.e., X0 vs. X3, X0 vs. X24, W0 vs. W3, and W0 vs. W24.

The relative changes in metabolism and the genes involved in phenylalanine metabolism are depicted in Figure 11. The results showed that a much greater number of genes encoding phenylalanine ammonia-lyase (PAL), cinnamate 4-hydroxylase (C4H), and 4-coumarate-CoA ligase (4CL) were responsible for the biosynthesis of the products of phenylalanine metabolism in X33 than in W7 under low-temperature stress. Except for the two common DEGs (Ibat. Brg.06A_G004160 and Ibat.Brag. 06D_G001990) encoding PAL in X33 and W7, four DEGs were uniquely expressed in X33. One DEG (Ibat. Brg.07D_G015300) and four DEGs encoding C4H at 3 h and 24 h of low-temperature stress treatment were detected in X33, respectively, while there were no DEGs encoding C4H in W7. Ibat.Brg.06B_G020670 was the only gene encoding 4CL in W7, while four DEGs encoded 4CL in X33. In addition, cinnamic acid and 4-coumaric acid are two important products of phenylalanine metabolism [42]. The contents of trans-cinnamate and p-coumaric acid were continuously increased in X33, while they increased first and then decreased in W7. The higher contents of DEMs in X33 may be associated with low-temperature stress tolerance in sweet potato.

### 3.9. Glutathione Metabolism in Response to Low Temperatures

**Figure 12:**
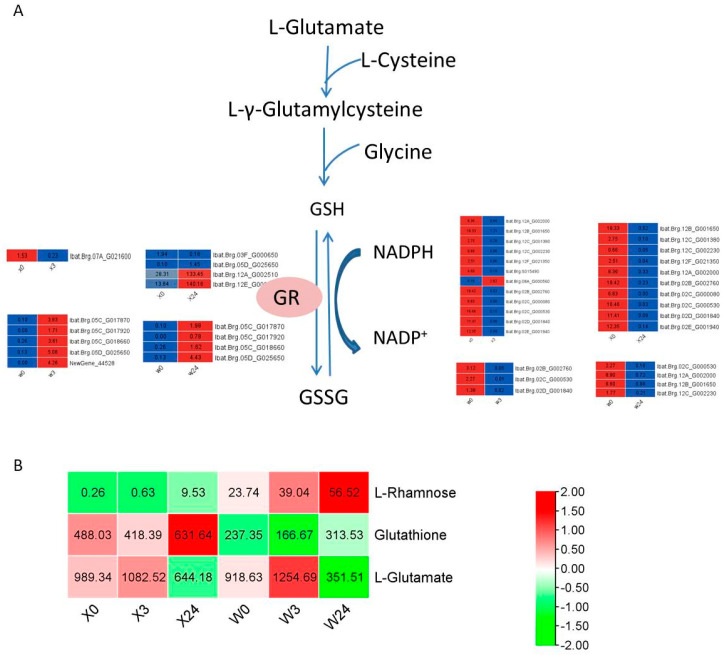
Adaptive changes involved in glutathione metabolism in X33 and W7 under low-temperature stress. (**A**) Heatmap of DEGs involved in glutathione metabolism. The solid arrow and dashed arrow represent one reaction and several reactions, respectively. The red and blue rectangles indicate the expression pattern (up-/downregulated) of DEGs based on the two comparisons, i.e., X0 vs. X3, X0 vs. X24, W0 vs. W3, and W0 vs. W24. (**B**) Heatmap of DEMs involved in glutathione metabolism. The red and green rectangles indicate the related contents (up-/downregulated) of DEMs based on the two comparisons, i.e., X0 vs. X3, X0 vs. X24, W0 vs. W3, and W0 vs. W24.

It was found that glutathione metabolism was significantly enriched in both metabolome and transcriptome data. Glutathione is synthesized from L-glutamate, L-cysteine, and glycine. As shown in Figure 12, although the L-glutamate and glutathione contents increased in both sweet potato varieties, the glutathione content in X33 was twice that in W7 at 24 h of low-temperature stress treatment. The DEGs encoding glutathione reductase (GR) differed greatly between the two sweet potato cultivars, indicating different glutathione metabolism pathways in response to low temperatures in X33 and W7.

## 4. Discussion

In the present study, two sweet potato genotypes (X33 and W7) were used as experimental materials for combined transcriptome and metabolism analysis. DEMs and DEGs were identified and their evolved metabolic pathways were deeply analyzed, yielding fresh insights regarding the low-temperature resistance mechanism of sweet potato.

The Ca^2+^ signature helps plants respond to low temperatures by mediating the Ca^2+^ concentration in the cytosol within a short time [43]. Calmodulins, plant phospholipases, CPKs/CDPKs, and CaM-like proteins trigger the Ca^2+^ cascade and transmit the cold stimulus via interacting with a range of downstream target proteins to adapt to low temperature [44]. CIPK is a type of serine/threonine-protein kinase that can specifically interact with CBLs and plays an important role in plant Ca^2+^ signaling. The model CBL1-CIPK7 regulates plant low-temperature response in Arabidopsis, and the overexpression of OsCIPK3 in rice and the ectopic expression of wheat TaCIPK14 in tobacco confer low-temperature tolerance [45,46,47]. Calmodulin 6 negatively regulates cold tolerance via attenuating ICE1-dependent stress responses in potato [48]. Seven calmodulins and several phospholipases were identified in this study that displayed upregulated expression patterns under low-temperature stress, which suggests that the Ca^2+^ signature helps sweet potato plants respond efficiently under low-temperature exposure. Mitogen-activated protein kinase (MAPK) proteins consist of three protein kinases (MAPKKK-MAPKK-MAPK) and play important roles in the regulation of cold response in plants via auto-phosphorylation, and the phosphorylation of various downstream stress-associated proteins [49]. The cascade MKK2-MPK4 is specifically activated by low-temperature stress, inducing the constitutively upregulated expression of CBF3 and enhancing low-temperature stress tolerance in Arabidopsis [50,51]. MPK3 and MPK6 are rapidly activated after low-temperature stress and negatively regulate the low-temperature response via promoting the degradation of ICE1 in Arabidopsis [52], whereas OsMAPK6 positively regulates the expression of OsDREB1 to transduce low-temperature signaling in rice [53]. As shown in Table 2, various TFs belonging to the CBF/DREB, NAC, MYB, WRKY, and bZIP gene families were involved in the low-temperature signaling pathway. These TFs may play vital roles in the regulation of low-temperature stress. A recent study reported that an ethylene-responsive factor (AP2/ERF) TF, OsERF52, acted as a positive modulator in response to low temperatures via interacting with OsICE1 [54]. Similarly, the homologous gene IbERF5 was found to be activated by low temperature, indicating that IbERF5 may be a candidate gene for further study. Additionally, the present study identified 12 hormone-related and 23 stress-related genes, which may contribute to low-temperature stress resistance in sweet potato.

The signaling link between cold responses and the clock function is complex. It was reported that several clock components, such as PRR5/7/9, TOC1, and EC can regulate CBFs expression, indicating that the clock output pathways apparently function upstream of the CBF-mediated cold response pathway [55]. In addition, cold-induced degradation of core clock proteins TIMING OF CAB EXPRESSION 1 implements temperature compensation in the circadian clock [56]. In our study, many DEGs were enriched in the “circadian rhythm-plants” pathway, proving the complexity of the signaling link between cold responses and the clock function.

Flavonoids protect cell membranes and proteins against low temperatures [57]. When plants suffer from low-temperature stress, the expression of flavonoid biosynthetic genes is upregulated and the content of flavonoids increases [58]. Flavonoids were introduced as potential biomarkers for cold stress in barley, further confirming the key role of flavonoids in enhancing cold resistance [59]. Three common DEMs belonging to flavonoids were detected between X33 and W7 at 0 h vs. 3 h, 0 h vs. 24 h, and 3 h vs. 24 h, respectively, including taxifolin, delphinidin 3-sophoroside, and gancaonin Q, which may help sweet potato plants counterbalance the excessive reactive oxygen species (ROS) production and repair the damage [60]. It has been reported that taxifolin can reduce the damage of tobacco PSII caused by low temperatures through multiple pathways, including antioxidation, photoprotection, and membrane stabilization [61]. As shown in Figure 9, 65 genes associated with taxifolin were identified in the present study. These genes may contribute to regulating the antioxidant oxidase system and repairing the damage induced by low temperatures.

Sugar metabolism, as a central process in energy supply and stress response, enhances plant cold resistance through osmotic regulation, energy provision, antioxidant defense, and signal transduction under low-temperature stress [38]. As a key energy regulation hub in plants under stress conditions, glycolysis enables rapid adjustments in energy allocation through modulating the activity of critical enzymes [62]. Hexokinase initiates glycolysis by catalyzing the first committed step of the reaction, and phosphofructokinase determines the overall rate of glycolysis [63,64]. Both enzymes dynamically regulate the glycolytic flux via sensing the cellular energy status (ATP/NADH levels), ensuring that the reaction proceeds irreversibly in one direction. In this study, the number and magnitude of DEGs encoding the two key enzymes differed between X33 and W7, as well as between time points. Most of the genes were downregulated by low temperature, which was consistent with previous reports in potato, demonstrating that low-temperature stress could slow the process of glycolysis via suppressing the activities of hexokinase and phosphofructokinase. Glucose-6-phosphate (G6P) is the first key intermediate in the glycolytic pathway. It can be converted into fructose-6-phosphate (F6P), thereby ensuring energy-yielding catabolism. G6P also participates in the PPP, providing NADPH (reducing power) and ribose-5-phosphate (a precursor for nucleic acid synthesis) [65]. Short-term low-temperature stress (3 h) promoted G6P synthesis in both X33 and W7, though its levels remained much higher in X33 than in W7. However, excessively high concentrations of G6P can inhibit hexokinase activity, thereby restricting carbon flux distribution [66]. After 24 h of low-temperature stress, the G6P content in W7 was much higher than that in X33, indicating that the glycolytic reaction in W7 was less active than in X33.

The phenylpropanoid pathway is a crucial metabolic route in plants, producing a variety of secondary metabolites that serve as precursors for downstream lignin and flavonoid biosynthesis [67]. Under low-temperature stress, lignin strengthens the cell wall to enhance cold resistance, while flavonoids act as antioxidants, mitigating ROS-induced damage [68]. The first three reactions in the phenylpropanoid pathway are catalyzed by phenylalanine ammonia-lyase (PAL), cinnamic acid 4-hydroxylase (C4H), and 4-coumarate-CoA ligase (4CL), generating intermediate metabolites such as cinnamic acid, p-coumaric acid, and p-coumaroyl CoA [69]. Numerous studies have demonstrated a strong correlation between the activity of these enzymes and low-temperature tolerance in plants [70,71,72]. These metabolites accumulated steadily in X33 but exhibited an initial increase followed by a decrease in W7, and the number of DEGs encoding these enzymes involved in the phenylpropanoid pathway was much higher in X33 than in W7 under low-temperature stress conditions. This finding implies that X33 can provide more raw materials than W7 for the downstream metabolism pathway, ensuring its stronger ability to resist low-temperature stress. Numbers of C4H were cloned from several plants with a highly conservative structural domain [73,74,75]. C4H genes are closely related to the lignification process in plants, and their activities affect multiple metabolites involved in the lignin synthesis pathway, such as coumaric acid, ferulic acid, sinapic acid, and caffeic acid [76]. The overexpression of C4H (CYP73A24) in transgenic tomato plants affected the flux into stem lignin and the accumulation of flavonoids [77]. GbC4H in ginkgo can be induced by low temperature [69], similar to the findings for X33. Interestingly, the number of DEGs encoding C4H was much higher in X33 than in W7, and the expression of this gene exhibited opposite patterns between the two sweet potato varieties. This result indicates that C4H genes may contribute to the improved low-temperature tolerance of sweet potato cv. X33.

GSH plays a characteristic role in mitigating ROS in the non-enzymatic antioxidant system, and the ratio of reduced GSH to oxidized glutathione (GSSG) (GSH/GSSG) is a key indicator of cellular redox status [70]. The ROS scavenging ability of GSH is an important mechanism to defend against low-temperature stress [78,79]. In the present study, the contents of GSH were increased in X33 and decreased in W7 under low-temperature conditions, suggesting that the improved low-temperature resistance of X33 was conferred by the higher GSH content. Taken together, the findings showed that compared to W7, X33 specifically accumulated certain metabolites related to carbohydrate metabolism, phenylpropanoid metabolism, and glutathione metabolic pathways under low-temperature stress. These metabolites may play unique roles in resistance to low-temperature stress in sweet potato, suggesting that X33 may possess a more complex and tightly regulated signaling network that responds to low-temperature stress via modulating metabolic changes.

Multi-omics analysis has promoted the development of molecular-assisted breeding, evolving from the traditional one-way selection of “genotype to phenotype” to a systematic design of “gene expression and dynamic metabolic regulation” to “agronomic trait formation”. Our research provided some candidate genes and some important regulatory pathways for molecular marker-assisted breeding. However, more experiments are needed to further elucidate the deeper mechanism of low-temperature stress resistance of sweet potato and achieve the breeding selection of low-temperature-resistant varieties.

## 5. Conclusions

In this study, the distinct DEGs and DEMs may result in the different levels of low-temperature stress tolerance between X33 and W7. The core genes involved in Ca^2+^ signaling, MAPK cascades, the ROS signaling pathway, and TF families (including bHLH, NAC, and WRKY) may play significant roles in the response to low temperature in sweet potato. Enrichment in the carbohydrate metabolism, phenylpropanoid metabolism, and glutathione metabolism pathways contributed to low-temperature tolerance in sweet potato, with the DEMs including flavonoids, soluble sugar, phenylpropanoids, and GSH. These results explain the differences between X33 and W7 in response to low-temperature stress, and the core DEGs and DEMs will be significant for revealing the mechanism of low-temperature resistance of sweet potato and promoting the development of breeding selection of low-temperature resistance in sweet potato.

## Figures and Tables

**Table 1:** Information on common differentially expressed metabolites (DEMs) in X33 and W7 exposed to low-temperature stress.

Metabolite ID	Metabolite Name	CAS ID	Molecular Formula	Molecular Weight (g mol^−1^)	Regulation in X33			Regulation in W7			KEGG		HMDB	
					X0_vs_X3	X0_vs_X24	X3_vs_X24	W0_vs_W3	W0_vs_W24	W3_vs_W24	Annotation	ko ID	ID	Taxonomy
neg_1840	Cistanoside A	N/A	C_36_H_48_O_20_	800.8	down	down	down	down	down	down	N/A	N/A	N/A	N/A
neg_2935	S-(5′-Adenosyl)-L-methionine	485-80-3	C_15_H_23_N_6_O_5_S^+^	399.4	up	up	up	down	up	up	N/A	N/A	HMDB0001185	5′-deoxyribonucleosides
neg_3962	4-Hydroxy-2,2′-bipyrrole-5-methanol	N/A	C_9_H_7_NO_2_	178.19	down	down	down	down	down	down	C21568	ko01100; ko01110	N/A	N/A
neg_4623	Oxprenolol	6452-71-7	C_15_H_24_NO_3_	265.35	down	down	down	down	down	down	N/A	N/A	HMDB0015520	Phenol ethers
neg_5034	Digoxigenin	N/A	C_41_H_64_O_14_	390.51	down	up	up	down	up	up	N/A	N/A	HMDB0060731	Steroids and steroid derivatives
neg_5512	Decanoyl-L-carnitine	1492-27-9	C_17_H_33_NO_4_	390.5	up	up	up	up	up	up	N/A	N/A	HMDB0000651	Fatty Acyls
neg_6852	1-Oleoyl-sn-glycero-3-phosphocholine	19420-56-5	C_26_H_52_NO_7_P	521.7	down	down	down	down	down	down	C03916	N/A	HMDB0002815	Glycerophospholipids
neg_6879	sesquicannabigerol	N/A	C_26_H_40_O_2_	384.6	up	down	down	up	up	down	N/A	N/A	N/A	N/A
neg_6937	(S)-N-Methylcoclaurine	N/A	C_18_H_21_NO_3_	299.4	down	down	down	down	down	down	C05176	ko00950; ko01100; ko01110	HMDB0060319	Isoquinolines and derivatives
neg_6983	yibeissine	N/A	C_27_H_41_NO_4_	443.6	down	down	down	down	down	down	N/A	N/A	N/A	N/A
pos_2008	N-Acetyldemethylphosphinothricin	N/A	C_6_H_12_NO_5_P	208.13	up	up	up	down	up	up	C17949	ko00440; ko01110	N/A	N/A
pos_2127	Taxifolin	N/A	C_15_H_12_O_7_	304.25	up	up	up	down	up	up	C01617	ko00941; ko01100; ko01110	HMDB0242509	Flavonoids
pos_2130	Delphinidin 3-sophoroside	59212-40-7	C_27_H_31_O_17+_	627.5	up	up	up	down	up	up	N/A	N/A	HMDB0038007	Flavonoids
pos_2764	Serratanidine	N/A	C_16_H_25_NO_4_	295.37	down	down	down	down	down	down	C09899	N/A	N/A	N/A
pos_3752	Gancaonin Q	134958-52-4	C_25_H_26_O_5_	406.5	down	down	down	down	down	down	N/A	N/A	HMDB0038875	Flavonoids
pos_3982	Cimicifugamide	N/A	C_25_H_31_NO_10_	505.5	down	down	down	down	down	down	N/A	N/A	N/A	N/A
pos_4068	Emindole SB	N/A	C_28_H_39_NO	405.6	down	down	down	up	down	down	C20527	ko01100; ko01110	N/A	N/A
pos_4232	Nicotianamine	N/A	C_12_H_21_N_3_O_6_	303.31	down	down	down	up	down	down	C05324	ko00270; ko00999; ko01100; ko01110	HMDB0255025	Carboxylic acids and derivatives
pos_5482	LysoPE 20:2	N/A	C_25_H_48_NO_7_P	505.6	down	down	down	down	down	down	N/A	N/A	N/A	N/A
pos_5703	5-Methoxyindoleacetate	3471-31-6	C_11_H_11_NO_3_	205.21	down	down	down	down	down	down	C05660	ko00380	HMDB0004096	Indoles and derivatives
pos_5743	Choline	62-49-7	C_5_H_14_NO^+^	104.17	down	down	down	down	down	down	C00114	ko00260; ko00564; ko01100; ko02010	HMDB0000097	Organonitrogen compounds
pos_6068	DG(18:4(6Z,9Z,12Z,15Z)/18:4(6Z,9Z,12Z,15Z)/0:0)	N/A	C_39_H_60_O_5_	608.9	up	up	up	down	up	up	N/A	N/A	HMDB0007338	Fatty Acyls
neg_4060	8-Demethyl-8-alpha-L-rhamnosyltetracenomycin C	N/A	C_28_H_28_O_15_	604.5	up	up	up	down	up	up	C20974	ko01100; ko01110	N/A	N/A
pos_2595	Aurachin B epoxide	N/A	C_25_H_33_NO_3_	395.5	up	up	down	up	up	down	C21874	ko01100; ko01110	N/A	N/A
pos_3724	6-Hydroxytryprostatin B	N/A	C_21_H_25_N_3_O_3_	367.4	down	down	down	down	down	down	C20513	ko01100; ko01110	N/A	N/A
pos_3802	Met His Phe	N/A	N/A	N/A	down	down	down	down	down	down	N/A	N/A	N/A	N/A
pos_5744	PC(18:1(9E)/0:0)[U]	N/A	N/A	N/A	down	down	down	down	down	down	N/A	N/A	N/A	N/A
pos_4028	Thr Cys Asn Ala	N/A	N/A	N/A	down	down	down	down	down	down	N/A	N/A	N/A	N/A
pos_4082	Glu Glu Glu	N/A	N/A	N/A	down	down	down	down	down	down	N/A	N/A	N/A	N/A
pos_4112	Asn Ser His Ser	N/A	N/A	N/A	down	down	down	up	down	down	N/A	N/A	N/A	N/A
neg_1665	2-Methyl-3-n-amyl-dihydropyrrole	N/A	N/A	N/A	up	up	down	up	up	down	C21571	ko01100; ko01110	N/A	N/A

**Table 2:** Information on common DEGs in Xu33 and W7 exposed to low-temperature stress.

Function Classification 1	Function Classification 2	Function Predication	Gene Numbers	Xu33			W7		
				X0 vs. X3	X0 vs. X24	X3 vs. X24	W0 vs. W3	W0 vs. W24	W3 vs. W24
Signal transduction mechanisms	Ca^2+^ signaling	calcium-binding protein KRP1-like	4	up	up	up	up	up	up
		calmodulin-binding protein 60 C-like isoform X1	1	up	up	up	up	up	up
		calmodulin-like protein 1	2	up	up	up	up	up	up
		putative calcium-binding protein CML19	3	up	up	up	up	up	up
	Kinases and Phosphatases	mitogen-activated protein kinase	3	up	up	up	up	up	up
		mitogen-activated protein kinase 3-like	6	up	up	up	up	up	up
		mitogen-activated protein kinase 9-like isoform X1	1	up	up	up	up	up	up
		mitogen-activated protein kinase kinase kinase 17-like	2	up	up	up	up	up	up
		probable protein phosphatase 2C 25	7	up	up	up	up	up	up
		ATP-dependent 6-phosphofructokinase 6-like	2	up	up	up	up	up	up
		serine/threonine-protein phosphatase 2A 57 kDa regulatory subunit B’ theta isoform-like	2	up	up	up	up	up	up
		serine/threonine-protein kinase AtPK2/AtPK19-like isoform X2	1	up	up	up	up	up	up
		G-type lectin S-receptor-like serine/threonine-protein kinase At4g27290 isoform X1	2	up	up	up	up	up	up
		inositol oxygenase 2-like	1	up	up	up	up	up	up
		inositol-3-phosphate synthase	2	up	up	up	up	up	up
		inositol-tetrakisphosphate 1-kinase 3-like	4	up	up	up	up	up	up
		probable inactive receptor kinase At5g10020	1	down	up	up	down	up	up
		probable LRR receptor-like serine/threonine-protein kinase At3g47570 isoform X1	1	down	down	down	down	down	up
		probable serine/threonine-protein kinase WNK11 isoform X2	1	up	up	up	up	up	up
		protein LYK5-like	1	up	up	up	up	up	up
		protein STRUBBELIG-RECEPTOR FAMILY 7-like	1	up	up	up	up	up	up
		rust resistance kinase Lr10-like isoform X1	1	up	up	up	up	up	up
Hormone-related	/	abscisic acid 8′-hydroxylase CYP707A2 [*Ipomoea triloba*]	6	up	up	up	up	up	up
		JA-domain [*Ipomoea batatas*]	1	up	up	up	up	up	up
		ethylene-responsive transcription factor 5 [*Ipomoea triloba*]	5	up	up	up	up	up	up
stress-related	pathogenesis-related	pathogenesis-related protein PR-4 [*Ipomoea triloba*]	2	up	up	up	up	up	down
	ROS-related	peroxiredoxin-2E-1, chloroplastic [*Ipomoea triloba*]	1	down	down	down	down	down	up
		peroxisomal (S)-2-hydroxy-acid oxidase [*Ipomoea triloba*]	1	down	down	down	down	down	up
		anionic peroxidase-like [*Ipomoea triloba*]	1	up	up	down	up	up	down
	cold-related	cold-responsive protein kinase 2-like [*Ipomoea triloba*]	1	up	up	up	up	up	up
		dehydration-responsive element-binding protein 1E-like	10	up	up	up	up	up	up
	aquaporin	aquaporin PIP1-2	6	down	down	down	down	down	down
		aquaporin PIP1	1	down	down	down	down	down	down
Transcription	AP2 domain	ethylene-responsive transcription factor 5	5	up	up	up	up	up	up
		dehydration-responsive element-binding protein 1E-like	10	up	up	up	up	up	up
	B3 DNA-binding domain	AP2/ERF and B3 domain-containing transcription repressor RAV2-like	1	up	up	up	up	up	up
	B-box zinc finger	zinc finger protein CONSTANS-LIKE 16	1	down	down	down	down	down	up
	bZIP transcription factor	bZIP transcription factor 53	1	up	up	up	up	up	up
	CCAAT-binding	nuclear transcription factor Y subunit A-1-like	1	up	up	up	up	up	up
	CCT motif	zinc finger protein CONSTANS-LIKE 16	6	up	up	up	up	up	up
	Dof domain, zinc finger	cyclic dof factor 2-like	5	up	up	up	up	up	up
	GRAS domain family	scarecrow-like protein 21	2	up	up	up	up	up	up
	Helix-loop-helix	transcription factor bHLH130-like	1	up	up	up	up	up	up
	HSF-type DNA-binding	heat shock factor protein HSF30	3	up	up	up	up	up	up
	mTERF	transcription termination factor MTEF1, chloroplastic	1	down	down	down	down	down	down
	Myb-like	protein CCA1-like isoform X1	2	up	up	up	up	up	up
		transcription factor HHO3-like	5	up	up	up	up	up	up
	No apical meristem (NAM) protein	NAC domain-containing protein 90-like	1	up	up	up	up	up	up
		NAC1-like protein	1	up	up	up	up	up	up
	two-component	two-component response regulator-like APRR5	2	up	up	up	up	up	up
	TAZ zinc finger	BTB/POZ and TAZ domain-containing protein 4	1	up	up	up	up	up	up
	TCP family	transcription factor TCP15-like	1	up	down	down	up	down	down
	WRKY	WRKY DNA-binding transcription factor 70-like	1	down	up	up	up	up	up
		probable WRKY transcription factor 40	2	up	up	up	up	up	up
		probable WRKY transcription factor 26 isoform X2	6	up	up	up	up	up	up

## Data Availability

Data are contained within the article or Supplementary Materials.

## Supplementary Materials

The following supporting information can be downloaded at: https://www.mdpi.com/article/10.3390/genes16080899/s1. Figure S1. Volcano plots for genes change analysis between different groups in X33 and W7.
