# Supplementary material for: Integrated Transcriptome and Metabolome Analysis Provides Insights into the Low-Temperature Response in Sweet Potato (Ipomoea batatas L.)
Source: Genes (Basel). 2025 Jul 28;16(8):899. doi: 10.3390/genes16080899 (PMC12385986; doi:10.3390/genes16080899)
Supplement: Supplementary file 1 [file genes-16-00899-s001.zip › genes-3755787-supplementary.pdf]

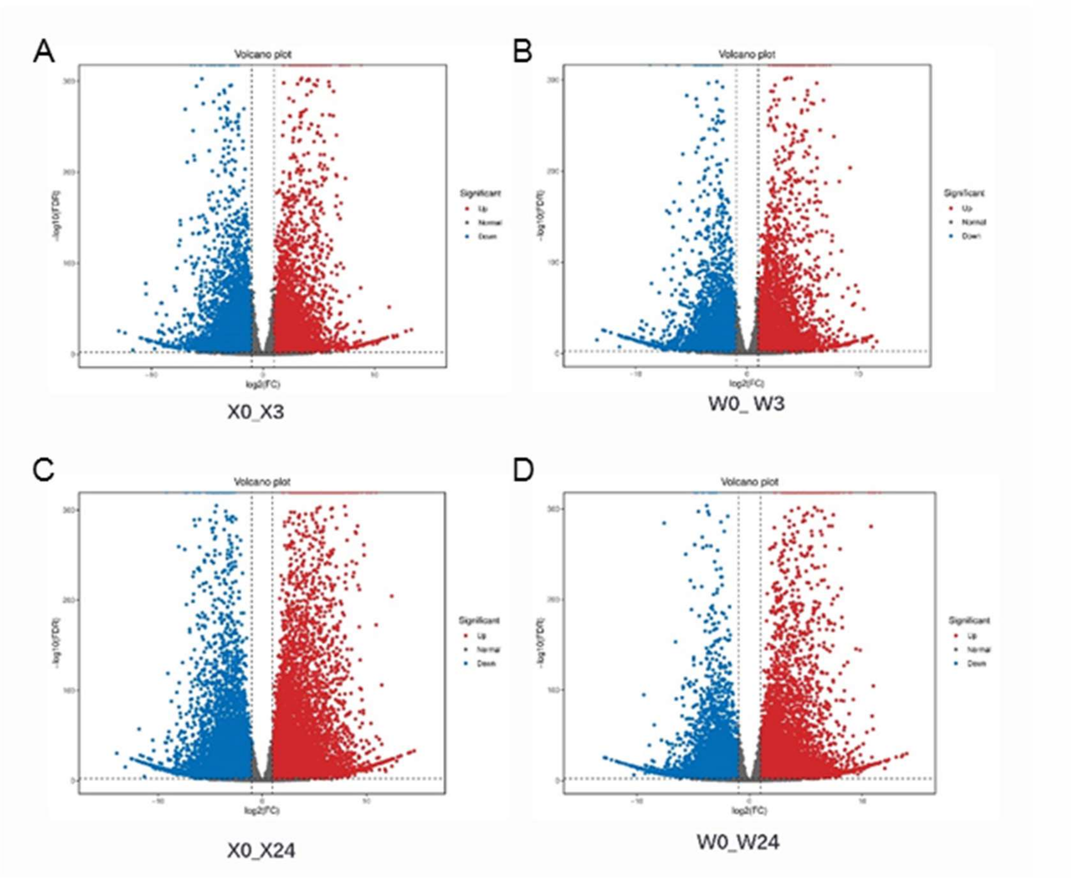

Supplement Figure S1. Volcano plots for genes change analysis between different groups in X33 and W7.

Volcano plots for genes change analysis between X0 vs. X3 (A), W0 vs. W3 (B), X0 vs. X24 (C), and W0 vs. W24 (D). X-axis presents $\log_2$ fold change ($\log_2$FC), showing the magnitude of differential expression. Y-axis present -$\log_{10}$(p-value), representing statistical significance. Red/blue points were significantly upregulated/downregulated genes and grey points were non-significant genes. The related description of methods and results have been added in the manuscript.
